# Experimentally measured methane hydrate phase equilibria and ionic liquids inhibition performance in Qatar’s seawater

**DOI:** 10.1038/s41598-020-76443-1

**Published:** 2020-11-10

**Authors:** M. F. Qureshi, M. Khraisheh, F. AlMomani

**Affiliations:** 1 Department of Chemical and Biomolecular Engineering, National University of Singapore, Singapore, Singapore; 2 Department of Chemical Engineering, Qatar University, Doha, Qatar

**Keywords:** Chemical engineering, Energy infrastructure

## Abstract

Qatar has the third-largest natural gas reserves in the world and is the second largest Liquefied natural gas (LNG) exporter in the world. These reserves are mainly located in its offshore North Field where the gas is extracted, transported to the onshore units, and is converted to LNG for international export. The formation of natural gas hydrates in the offshore subsea lines can cause unwanted blockages and hinder the smooth supply of gas supply from offshore to onshore units. In the present work, the formation and dissociation of methane gas hydrates have been studied in the ultra pure water system (UPW), artificial seawater (ASW), and Qatar seawater (QSW) at different conditions (4–10 MPa) using standard rocking cell rig. The naturally occurring seawater was collected from *Ras Laffan* seacoast located in Doha, Qatar. The seawater sample was examined for elemental analysis (SO_4_, Cl, Na, Ca, Mg, K, and Fe) using inductively coupled plasma atomic emission spectroscopy (ICP-AES) technique and its other properties like density, electrical conductivity, and pH were also measured. The experimental results show that the CH_4_ pure water HLVE curve is suppressed by about 3 K in Qatar seawater and 2 K in artificial seawater. The hydrate inhibition strength of the Ionic liquids (ILs) salts 3-Ethyl-1-methyl-1H-imidazol-3-ium methane-sulfonate [C_7_H_14_N_2_O_3_S] and 3-Ethyl-1-methyl-1H-imidazol-3-ium dicyanoazanide [C_8_H_11_N_5_] was evaluated in both the ultra pure water and Qatar seawater systems. Their performance was compared with methanol and other ILs salts reported in the literature. The selected ILs exhibited poor hydrate inhibition effect in the ultra pure water systems, but they show a noticeable thermodynamic and kinetic hydrate inhibition effect in the Qatar seawater system. The computational 3D molecular models of ILs and methanol were generated to cognize the plausible hydrate inhibition mechanism in the presence of these inhibitors.

## Introduction

Gas hydrates are crystalline solid compounds that are formed when small molecules like methane and ethane get trapped within water molecules under high pressure and low-temperature conditions^1^. The naturally occurring gas hydrates are a great source of energy^2^ that can store gas at high density, but the formation of gas hydrates in the subsea pipelines and deepwater drilling equipment post a serious threat to offshore flow assurance as they can lead to unwanted blockages in offshore subsea lines and interrupt the offshore operations^3–5^. Therefore, it's essential to have effective hydrate mitigation for smooth and safe offshore operations for oil and gas sector^6,7^.

Qatar is reported to possess the third-largest natural gas reserves in the world and is also the largest exporter of the liquefied natural gas in the globe^8^. The LNG trade demands a consistent supply of gas from offshore to onshore units and the formation of gas hydrates can hinder this supply. The water that exists in the subsea lines is likely to be saline in nature, it’s essential to understand the characteristics of hydrate formation and dissociation dynamics in the actual seawater system to develop an effective hydrate mitigation strategy, and select the right set of chemical hydrate inhibitors^9^. Generally, the chemical hydrate inhibitors are classified as thermodynamic hydrate inhibitors (THI)^10^, kinetic hydrate inhibitors (KHI)^11^, and anti-agglomerates (AA)^12^. Thermodynamic hydrate inhibitors like methanol and mono-ethylene glycol are widely used in the industry for hydrate prevention^10^. These hydrate inhibitors function well, but they are required in bulk quantities (> 30 wt%)^13^, and there are environmental and safety concerns associated with the disposal of these chemicals^14^. Therefore, over the last decade, the research interest has shifted towards the KHI that required in much lower quantity (≤ 5 wt%)^15^. The Ionic liquids (ILs) are a new class of low dosage hydrate inhibitors (LDHI) that have been already used for carbon capture^16,17^, wastewater treatment^18^, desalination^19,20^, and gas separation^21^. The ILs offer a potential substitute for carbon capture and storage because of their negligible vapor pressure and high thermal stability, which reduces solvent losses^22,23^. Some classes of ILs are reported to be environmentally friendly and tend to act as both THI and KHI^24–30^. By making simple changes in the structure of anion or the cation, properties like solubility, density, reflective index and viscosity of ILs can be adjusted to suit the process requirements^31,32^. This makes them an ideal inhibitor to be used for hydrate prevention and other processes. In addition, they can be designed for a special application by tuning of cation, anion and functional groups^33,34^. However, there are main concerns about their economic feasibility on large commercial scale and now some research is being diverted towards other compounds like amino acids^35^.

Rouher and Barduhn^36^, were among the first groups to work with seawater and they published gas hydrate equilibrium data in seawater to design a desalination process using iso-butane hydrates. Ohgaki et al.^37^, studied the CO_2_ hydrate formation in the seawater and the pure water. Englezos and Bishnoi^38^, developed a model for predicting water activity and methane hydrate stability conditions in artificial seawater. Similarly, Dholabhai et al.^39^ studied hydrate formation and equilibrium conditions in synthetic seawater using methane, propane, and carbon dioxide. At different salinity levels Tishchenko et al.^40^, developed a model for predicting methane hydrate equilibrium curves. The methane hydrate dissociation conditions with NaCl solutions was experimentally demonstrated by Maekawa et al.^41^ Whereas, Dickens and Quinby‐Hunt^42^, reported the methane hydrate stability conditions in the seawater with the salinity of up to 33.5% and observed that the methane hydrate dissociation temperature is depressed by almost—1.1 °C in the seawater system compared to the pure water system. Yang and Xu^43^, studied the effect of salinity on methane gas hydrate system and their numerical modeling results show that the methane hydrate zone gets shallower with the rise in the salinity and the stability of hydrate crystals is also reduced. In addition to that, they also stated that the presence of some salts in the seawater system can also promote the actual hydrate crystal formation in the hydrate stability zone. Li et al.^44^, reported phase equilibrium conditions of CH_4_ hydrate in 3.5 wt% NaCl, KCl, CaCl_2_ and MgCl_2_ solutions experimentally investigated at the temperature–pressure ranges of 281.9–287.1 K and 7.33–14.02 MPa. They also proposed a model based on the Chen–Guo model and the Hu–Lee–Sum (HLS) correlation for hydrate prediction. Their experimental outcomes show that the inhibition effect of MgCl_2_ is more significant than that of other salts. Hu et al.^45^, investigated the aptness of the Hu–Lee–Sum (HLS) correlation to calculate structure I hydrates and structure II hydrates depression temperature in any single salt or mixed salt-gas systems. According to them, the Hu–Lee–Sum (HLS) correlation, with introduction of certain parameters, is able to predict the hydrate depression temperature for complex gas and salt mixtures. Khan et al.^46^, carried out hydrate formation experiments using methane + ethane (74.7/25.3 mol%) gaseous mixtures and indicated that the formation onset times were three times longer in salt water compared to fresh water. Husebø et al.^47^ reported, that the hydrates stability in the reservoir relies on various factors which include interaction between minerals and nearby fluids. Normally, the salinity level increases with the depth in the reservoir and the formation of hydrate in the saline environment may increase the salinity level of the fluid surrounding the formed hydrate^47^. Due to this factor, the hydrates formed in the saline environment tend to be very non-uniform and may also lead to liquid pockets of the residual aqueous solution with high salinity^47^.

In this work the CH_4_ hydrate dissociation points in ultra pure water system (UPW) was compared with CH_4_ hydrate dissociation points in artificial seawater (ASW), Qatar seawater (QSW) and other saline water systems reported in the literature. The CH_4_ gas was chosen for easier comparison with literature data. The novelty aspect of this work is the use of Qatar seawater to experimentally investigate CH_4_ hydrate formation and dissociation at diverse conditions (4–12 MPa). The hydrate inhibition performance of the Ionic liquids (ILs) salts C_7_H_14_N_2_O_3_S and C_8_H_11_N_5_ was evaluated in the ultra pure water system and Qatar seawater systems at different pressure conditions (4–12 MPa) and their performance was compared with industrial thermodynamic inhibitor methanol and other ILs salts reported in the literature. All the experiments were conducted in the standard rocking cell rig (RC-5) installed at the Qatar University. The 3D computational models of selected ILs and methanol were generated to conceptualize the plausible inhibition mechanism.

## Experimental section

### Material and samples

The pure methane (CH_4_) gas with a purity of 99.9% was purchased from Buzware Scientific and Technical Gases, Doha (Qatar). The list of ILs and other chemicals used for the experiments is shown in Table 1. These chemicals were purchased from Sigma Aldrich USA and IoLiTec Germany with purity (> 98%). The fresh seawater sample was obtained from the bay closer to the Ras Laffan Industrial City Qatar. The sample was analyzed for pH, EC, and element detection using Thermo Orion Versa Star Pro Multiparameter Benchtop Meter, Thermo Scientific iCAP 6500-ICP-OES Spectrometer, respectively. The elemental content analysis of the seawater sample is shown in Table 2. All the ILs sample solutions were prepared with ultra pure water at room temperature using an electronic balance with a precision of ± 0.0001 g^48^.

**Table 1:**
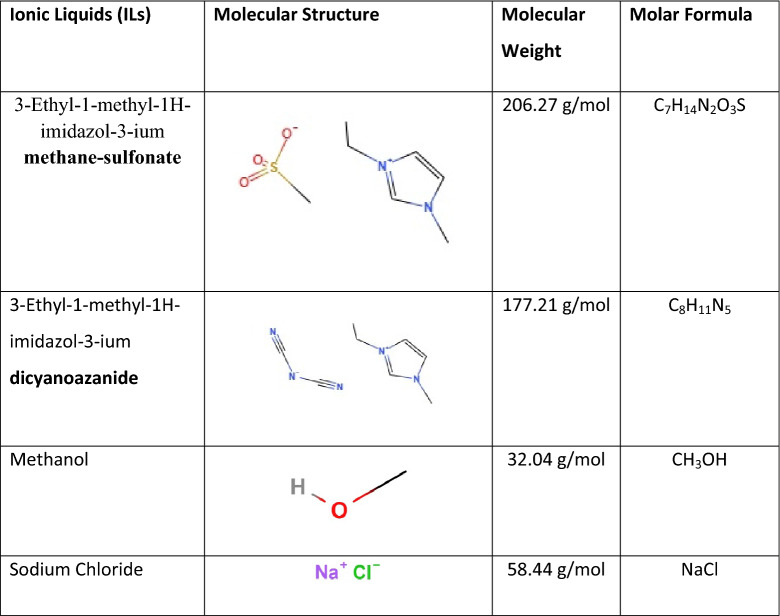
List of Ionic Liquids (ILs) used and their structures.

**Table 2:** Content analysis of Qatar sea water sample.

Test	Result	Unit
pH	8.05	
EC	64.93	mc/cm
SO_4_	1500	ppm
Cl	19,370	ppm
Na	13,352	ppm
Ca	484	ppm
Mg	1477	ppm
K	449	ppm
Fe	8.76	ppm
Sr	8.96	ppm

### High-pressure apparatus and procedures

**Figure 1:**
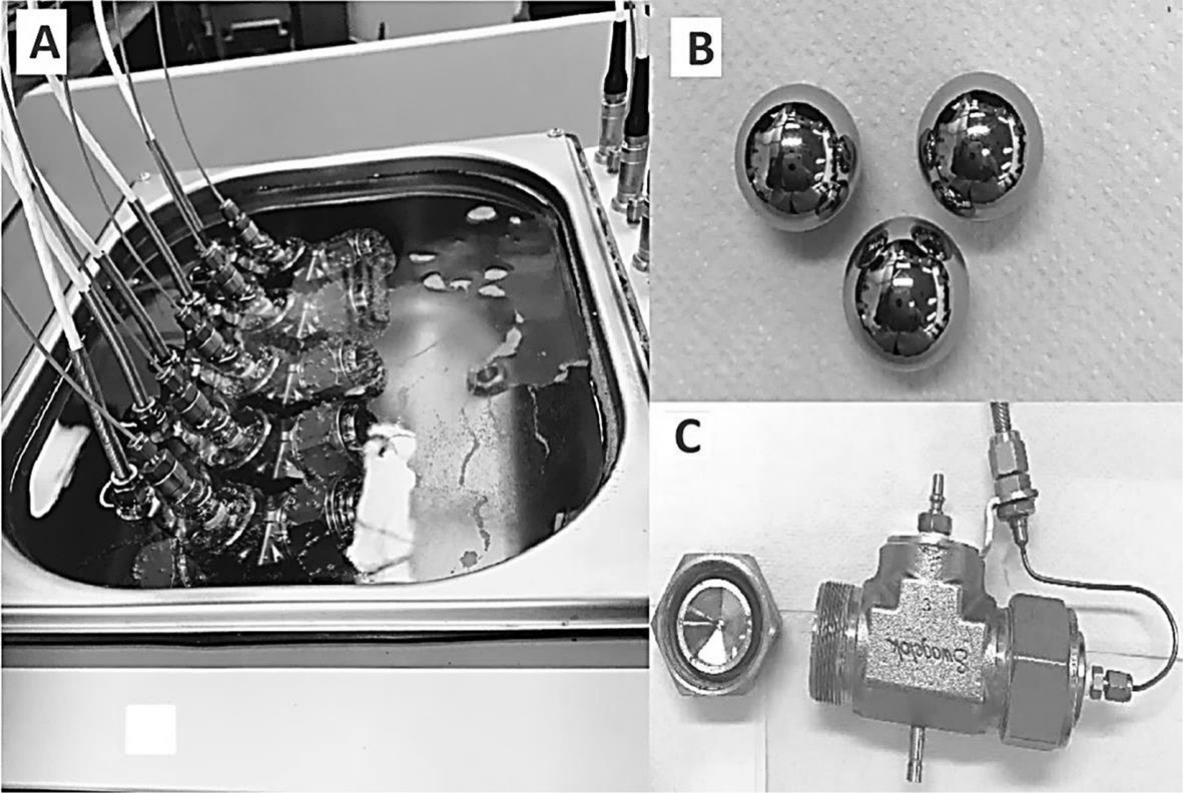
(**A**) Rocking Cell (RC-5) assembly used for the experiment, (**B**) stainless steel balls for agitation, and (**C**) stainless steel cell with screw cap.

**Figure 2:**
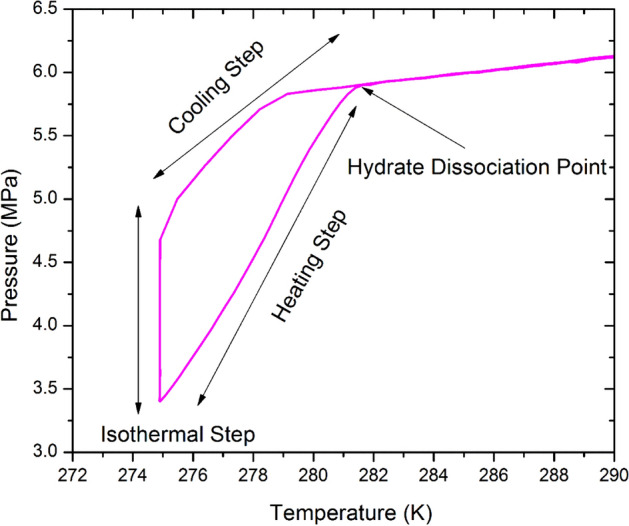
The experimental loop for a sample showing three experimental steps and the hydrate dissociation point.

**Figure 3:**
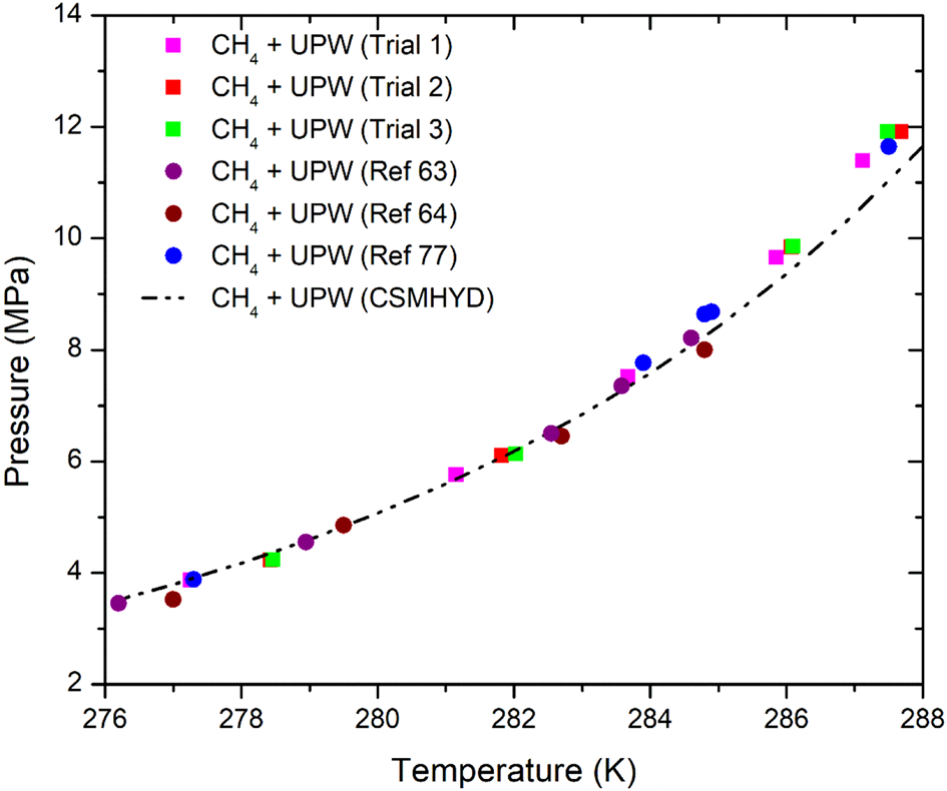
The repeated experimental trials for CH_4_ pure water HLVE and comparison of the ultra pure water experimental HLVE data with literature data Refs.^63,64,77^ and the data is also compared with hydrate simulation package CSM Hydrate^61^. A good agreement was observed between experimental, literature and CSM Hydrate package data^61^.

The methane phase equilibria (HLVE) or hydrate dissociation points were obtained in ultra pure water system (UPW), artificial seawater (ASW) and Qatar Seawater (QSW) using the standard rocking cell assembly (RC-5) provided by PSL SystemtechniK GmbH (Fig. 1), located in Chemical Engineering lab at Qatar University in 2018 (Tables 3, 4 and 5). It consists of 5 stainless steel (AISI 316L) made cells that are attached to the same skid that rocks the cells and are operated simultaneously. The system is able to bear up to 20 MPa pressure and within the temperature range of −263 to 333 K. The temperature sensors have the accuracy of ± 0.01 °K and pressure sensors have an accuracy of 0.1%. The combined standard uncertainty for the experiments was found to be less than < 1%. The temperature and pressure values with time were recorded during the experiment run with help of pre-installed specialized software by PSL. A diluted inhibitor solution of sample 15 cm^3^ was added to each cell and the cells were carefully tightened and placed on a rocking skid that was submerged in a cooling bath. Then the loaded cells, immersed in the bath, were cooled from 293.15 to 275.15 K within 9 h at the cooling rate of 1.8 K per h. This step is followed by an isothermal step of 24 h at a fixed temperature of 275.15 K and then finally the cells were slowly heated at the rate of 0.1 K per h till the temperature reached back to 293.15 K (Fig. 2)^49^. As the temperature is increased further the hydrate phase completely disappears. To maintain the accuracy experiments were performed 2–3 times to ensure the reliability of the results with different pressures by using the same protocol. The overall pressure drop^50^ during hydrate formation was monitored classically using pressure sensors. Figure 3**,** shows the repeated experimental trials for CH_4_ HLVE and comparison of the experimental data with literature and simulation data. Table 3, shows the CH_4_ HLVE data points obtained using the rocking cell assembly for three experimental trials. The experimental results were found to be in good agreement with the literature data and the standard error in the repeated experiments was about 0.05 K. More experimental procedures and equipment details have been reported in our previous works^13,35,51–55^.

**Table 3:** Pure methane HLVE (hydrate liquid–vapor equilibrium) data points for three experimental trials.

Trial 1		Trial 2		Trial 3	
T (K)	P (MPa)	T (K)	P (MPa)	T(K)	P(MPa)
287.12	11.40	287.68	11.91	287.48	11.92
285.85	9.66	286.07	9.85	286.09	9.86
283.67	7.52	281.82	6.10	282.02	6.13
281.15	5.76	278.42	4.23	278.46	4.24
277.25	3.87

**Table 4:** CH_4_ HLVE (hydrate liquid–vapor equilibrium) data points obtained in the ultra pure water and Qatar seawater sample solutions in the presence of 5wt% ionic liquid salts and 5wt% methanol.

Components	Ultra pure water system		Qatar sea water	
	T (K)	P (MPa)	T (K)	P (MPa)
CH_4_	287.12	11.4	285.93	12.78
	285.85	9.658	284.36	11.21
	283.67	7.518	282.15	9
	281.14	5.763	279.74	6.59
	277.24	3.87		
CH_4_ + 5wt% C_7_H_14_N_2_O_3_S	287.16	11.77	285.31	11.23
	285.7	9.83	283.92	9.42
	283.8	7.89	281.71	7.71
	281.28	5.89	280.98	6.74
	278	4.1	279.57	5.72
CH_4_ + 5wt% C_8_H_11_N_5_	286.91	11.40	284.84	11.29
	285.69	9.78	283.31	9.41
	283.64	7.76	282.11	7.83
	281.53	5.98	279.45	5.67
	277.71	3.99		
CH_4_ + 5wt% MeOH	283	8.86	282.93	11
	282	7.96	281.92	9.48
	280	6.45	280.06	7.50
	278	5.26	277.90	5.73

The HLVE data for 5wt% methanol in pure water system was obtained using CSMHYD^61^.

**Table 5:** CH_4_ HLVE (hydrate liquid–vapor equilibrium) data points experimentally obtained in the artificial sea water (ASW) (5 wt% NaCl).

T (K)	P (MPa)
285.22	11.16
284.25	10.01
281.91	7.81
281.01	6.75
279.69	5.97

### Extraction of gas hydrate dissociation and induction points

The extraction of accurate equilibrium point was conducted using the previously reported method by Tohidi et al.^56^. The gas hydrate dissociation point is extracted when the H–V (hydrate–vapor) and the H–L_w_–V (hydrate–liquid–vapor) equilibrium lines intersect with each other^57^. The point noted where the two equilibrium points intersect that is called ‘hydrate dissociation point’ as illustrated in Fig. 2^57^. For kinetics, the hydrate formation or induction point is an interval where the first hydrate crystal sighted (formation) by a sharp pressure drop during the cooling process of the heating–cooling cycle^53^. The hydrate induction times were calculated using the pressure–time (P–t) curves plotted for a specific sample using the recorded experimental data. The hydrate induction has been defined in different ways based on their measurement techniques^58,59^. In this work, the hydrate induction or formation time is taken as the point where a sharp pressure decline occurs^60^. The detailed calculation procedure for the hydrate induction time has been reported in previous studies^52,54^. The uncertainty in the reported induction times is within the range of ± 0.3 h (20 min). The temperature and pressure sensors were calibrated and the reliability of the results was checked by repeating the experiments (Table 2) and comparing the experimental data for the pure methane hydrate liquid–vapor equilibrium (HLVE) curve with the literature values and the simulation results (Fig. 3).

## Results and discussion

Initially, the pure methane (CH_4_) hydrate dissociation points (thermodynamics) and induction times (kinetics) were evaluated in a ultra pure water system at different pressure conditions (4–10 MPa). Then the same experiments were conducted using pure methane in the artificial seawater and Qatar seawater sample solutions at similar pressure conditions. Then the thermodynamic and kinetic hydrate inhibition effect of Ionic liquid (IL) salts and methanol was investigated in the ultra pure water and Qatar seawater systems both. Table 4, shows CH_4_ HLVE (Hydrate Liquid–Vapor Equilibrium) data points obtained in the ultra pure water and Qatar seawater sample solutions in the presence of 5wt% ionic liquid salts and 5wt% methanol. The HLVE data for pure water system in presence of 5wt% methanol was obtained using CSMHYD^61^. Table 5**,** Shows CH_4_ HLVE data points experimentally obtained in the Artificial Sea Water (ASW) (5 wt% NaCl).

### Thermodynamic hydrate inhibition (THI)

**Figure 4:**
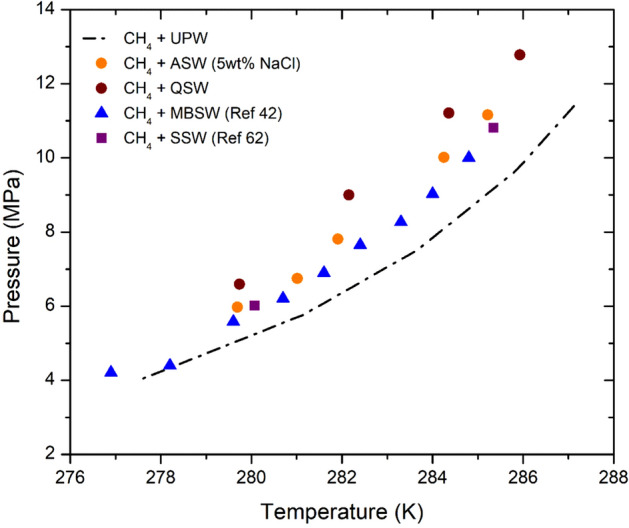
Comparing CH_4_ HLVE curve in ultra pure water (UPW) with artificial seawater (ASW), Qatar sea water (QSW), Monterey Bay (off the California coast US) Sea Water (MBSW)^42^ and Synthetic Sea Water (SSW)^62^. The CH_4_ pure water HLVE curve was suppressed by 3 K in QSW, 2 K in ASW, 1.5 K in SSW and 1 K in MBSW. The experimental HLVE data of this work has the uncertainty of about ± 0.05 K.

The thermodynamic analysis (Fig. 4) of ultra pure water and seawater systems indicate that the hydrate dissociates at low-temperature conditions in the seawater systems compared to the ultra pure water system at similar pressure conditions. The ultra pure water CH_4_ HLVE curve was suppressed by 3 K in Qatar seawater and 2 K in artificial seawater (5 wt% NaCl). The ultra pure water CH_4_ HLVE results were also compared with the Monteray Bay (Off the California coast) seawater (MBSW) CH_4_ HLVE results (S = 33.4%) reported by Dickens and Quinby‐Hunt^42^ and synthetic seawater (SSW) CH_4_ HLVE results (S = 36.6%, NaCl = 2.43 wt %) reported by Atik et al.^62^. The ultra pure water CH_4_ HLVE curve was suppressed by 1 K in MBSW and suppressed by 1.5 K in SSW. Hence, the higher salinity levels may aid in suppressing the hydrate dissociation conditions. These results indicate that the development of a hydrate mitigation strategy based on tests or modeling work conducted merely on a pure water system is not sufficient. The test can be preferably conducted on the actual offshore seawater samples to get a more reliable data set.

### THI effect of Ionic liquids in the pure water system and Qatar seawater system

**Figure 5:**
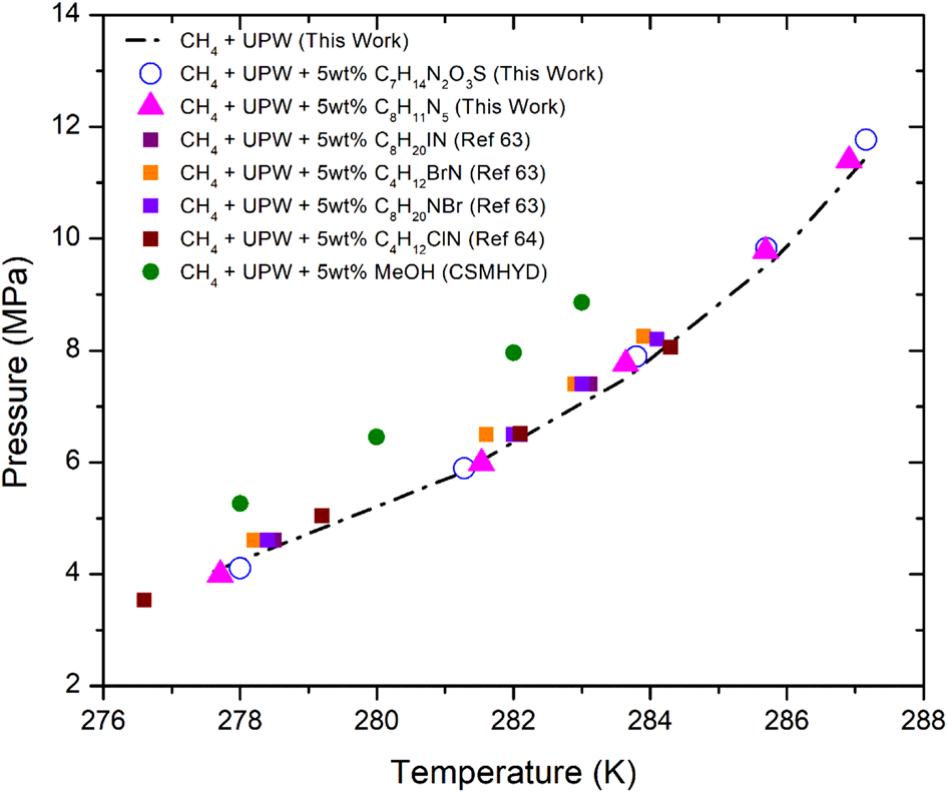
Shift in the ultra pure water CH_4_ HLVE in the presence of 5wt% IL salts 3-Ethyl-1-methyl-1H-imidazol-3-ium-methane sulfonate [C_7_H_14_N_2_O_3_S], 3-Ethyl-1-methyl-1H-imidazol-3-ium-dicyanoazamide [C_8_H_11_N_5_], Tetra-ethyl-ammonium Iodide [C_8_H_20_ IN]^63^, Tetra-ethyl-ammonium Bromide [C_8_H_20_ NBr]^63^, Tetra-methyl-ammonium Bromide [C_4_H_12_ BrN]^63^, Tetra-methyl-ammonium Chloride [C_4_H_12_ ClN]^64^ and Methanol [MeOH]^61^. The experimental HLVE data of this work has the uncertainty of about ± 0.05 K.

The THI strength of the selected Ionic liquids (ILs) salts, 3-Ethyl-1-methyl-1H-imidazol-3-ium methane-sulfonate [C_7_H_14_N_2_O_3_S] and 3-Ethyl-1-methyl-1H-imidazol-3-ium dicyanoazanide [C_8_H_11_N_5_] was evaluated in both the ultra pure water and Qatar seawater systems. In a ultra pure water system, their inhibition performance was compared with methanol and other ammonium ILs salts, Tetra-ethyl-ammonium Iodide [C_8_H_20_ IN], Tetra-ethyl-ammonium Bromide [C_8_H_20_ NBr], Tetra-methyl-ammonium Bromide [C_4_H_12_ BrN], Tetra-methyl-ammonium Chloride [C_4_H_12_ ClN] reported by Qasim et al.^63^ and Khan et al.^64^. As shown in Fig. 5, the 5wt% ILs salts used in this work and those reported in literature showed no significant THI effect and in comparison, 5wt% methanol was able to shift the CH_4_ HLVE by about 2 K at all pressure conditions. This indicates that in the case of ILs salts the 5 wt% dosage may not be sufficient enough to see a significant THI effect. Thus, higher concentration or dosage of ILs salts may be required to enhance their THI effect.

Stark et al.^65^, studied the binary system of water and IL 3-Ethyl-1-methyl-1H-imidazol-3-ium methane-sulfonate [C_7_H_14_N_2_O_3_S]. They observed that the water molecules do not tightly bound with the selected IL [C_7_H_14_N_2_O_3_S]. Their activation energy analysis indicates that there exists a barrier that hinders the translational motion or self-diffusion between ionic liquids anion, cation, and water molecules. This may be preventing the bidding of free water molecules by the IL [C_7_H_14_N_2_O_3_S].

**Figure 6:**
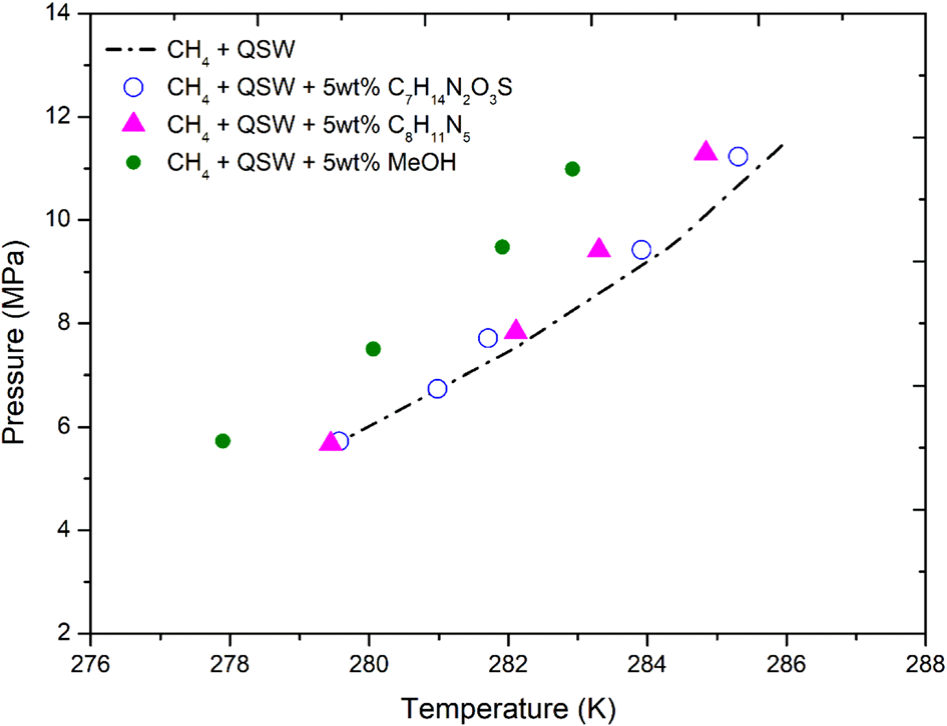
Shift in the Qatar seawater CH_4_ HLVE in the presence of 5wt% IL salts 3-Ethyl-1-methyl-1H-imidazol-3-ium-methane sulfonate [C_7_H_14_N_2_O_3_S], 3-Ethyl-1-methyl-1H-imidazol-3-ium-dicyanoazamide [C_8_H_11_N_5_] and Methanol [MeOH]. The experimental HLVE data of this work has the uncertainty of about ± 0.05 K.

**Figure 7:**
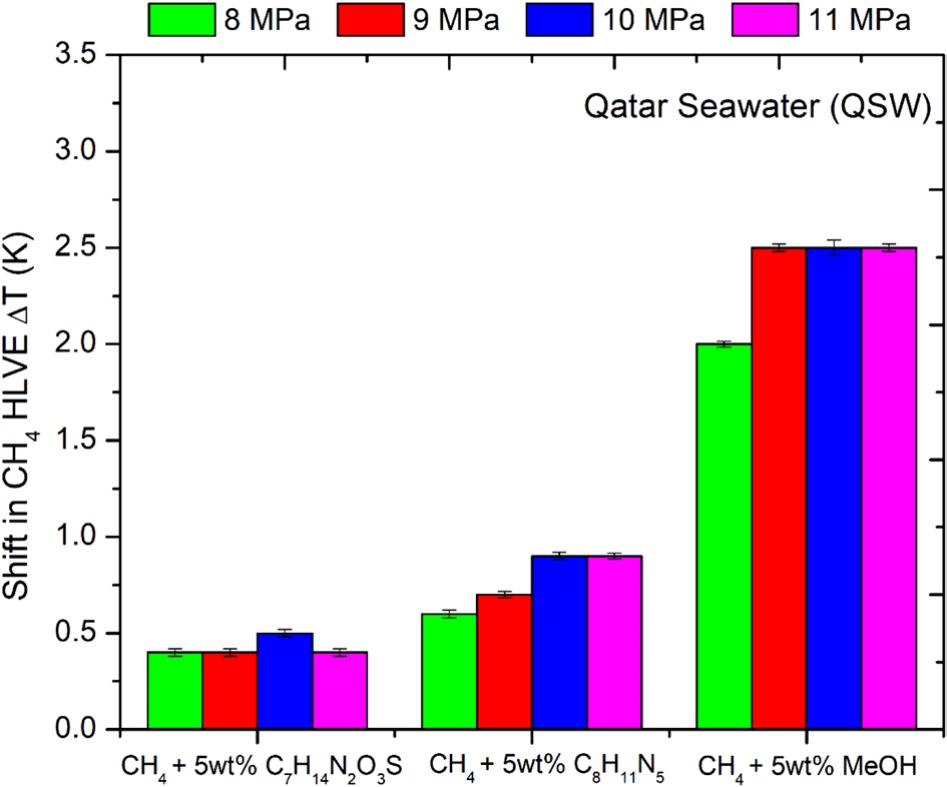
Shift in CH4 HLVE (Qatar seawater) in the presence of 5wt% IL salts 3-Ethyl-1-methyl-1H-imidazol-3-ium-methane sulfonate [C_7_H_14_N_2_O_3_S], 3-Ethyl-1-methyl-1H-imidazol-3-ium-dicyanoazamide [C_8_H_11_N_5_] and Methanol [MeOH]. The standard deviation of 0.01–0.05 K was calculated for the above data set. The above data has the uncertainty of about ± 0.05 K.

The same experiments with selected 5wt% ILs and 5wt% methanol were repeated using the seawater system containing the SO_4_^2−^, Cl^−^, Na^+^, and Mg^2+^ ions. As shown in Fig. 5, in the ultra pure water system the ILs showed no THI effect. However, in the Qatar seawater system with the dissolved ions the ILs shifted the CH_4_ HVLE within the range of 0.4–1.0 K at the pressure range of 4–12 MPa (Figs. 6, 7). Del Villano and Kelland^66^, reported that some ILs are weak hydrate inhibitors, but both can act as good synergist in the presence of other commercial kinetic inhibitors or salts. Thus, the selected ionic liquids were observed to be weak thermodynamic inhibitors but may tend to act as the synergist in the presence of SO_4_^2−^, Cl^−^, Na^+^, and Mg^2+^ ions. The salt and ions present in the seawater can also act as hydrate inhibitor themselves and affect the thermodynamic stability of natural gas hydrate. The ions present in the aqueous solution may reduce the chemical potential of liquid water leading to the prevention of hydrate formation.

As shown in Fig. 7, the IL [C_7_H_14_N_2_O_3_S] showed a similar THI effect at variable pressure conditions (8–11 MPa) and provided the temperature shift of about 0.4–0.5 K (± 0.05 K). In comparison, the IL [C_8_H_11_N_5_] provided a better THI effect and provided a temperature shift of about 0.6–0.9 K (± 0.05 K) within the pressure range of 8–11 MPa. The maximum temperature shift of about 0.9 K was provided by IL [C_8_H_11_N_5_] at higher pressures of about 10 MPa. Thus, the IL [C_8_H_11_N_5_] was observed to be slightly more effective than the IL [C_7_H_14_N_2_O_3_S]. In comparison, the methanol [CH_3_OH] provided the temperature shift of about 2–2.5 K (± 0.05 K). Thus in terms of effectiveness the inhibitors can be listed as: CH_3_OH > C_8_H_11_N_5_ > C_7_H_14_N_2_O_3_S .

According to You et al.^67^, methane hydrate formation and dissociation are affected by salinity in a closed system. In a closed saline system, during hydrate formation, the salts are separated from hydrate which leads to an increase in the salinity levels of the surrounding fluid. This causes the system to be separated into three equilibrium phases (gas, water, and hydrate phases) and impedes the further formation and growth of hydrate crystals. Saw et al.^68^, investigated the methane hydrate formation and dissociation in the synthetic seawater and observed that the hydrate formation and dissociation strongly rely on the salinity level of the chosen water sample. The hydrate dissociation pressure tends to increase with the increase in the salinity of the synthetic seawater. According to Saw et al.^68^, the solubility of methane in the aqueous solution tends to decreases with the increase in the level of salinity may be due to an increasing amount of interactions between dissolved ions and the guest CH_4_ molecule.

### Kinetic hydrate inhibition (KHI)

The hydrate induction time is considered to be a good indicator for evaluating the kinetic inhibition strength of the hydrate inhibitors^69^. The hydrate formation time may vary from seconds to days as a result of the complex nature of the hydrate formation process^69^. According to Sloan Jr and Koh^3^, the hydrate induction time relies on different factors, which include subcooling temperature, water history, impurities, gas composition, and system geometry. Therefore, the hydrate kinetic experiments cannot be replicated and the results obtained from one system may not match with the results of the other system.

**Figure 8:**
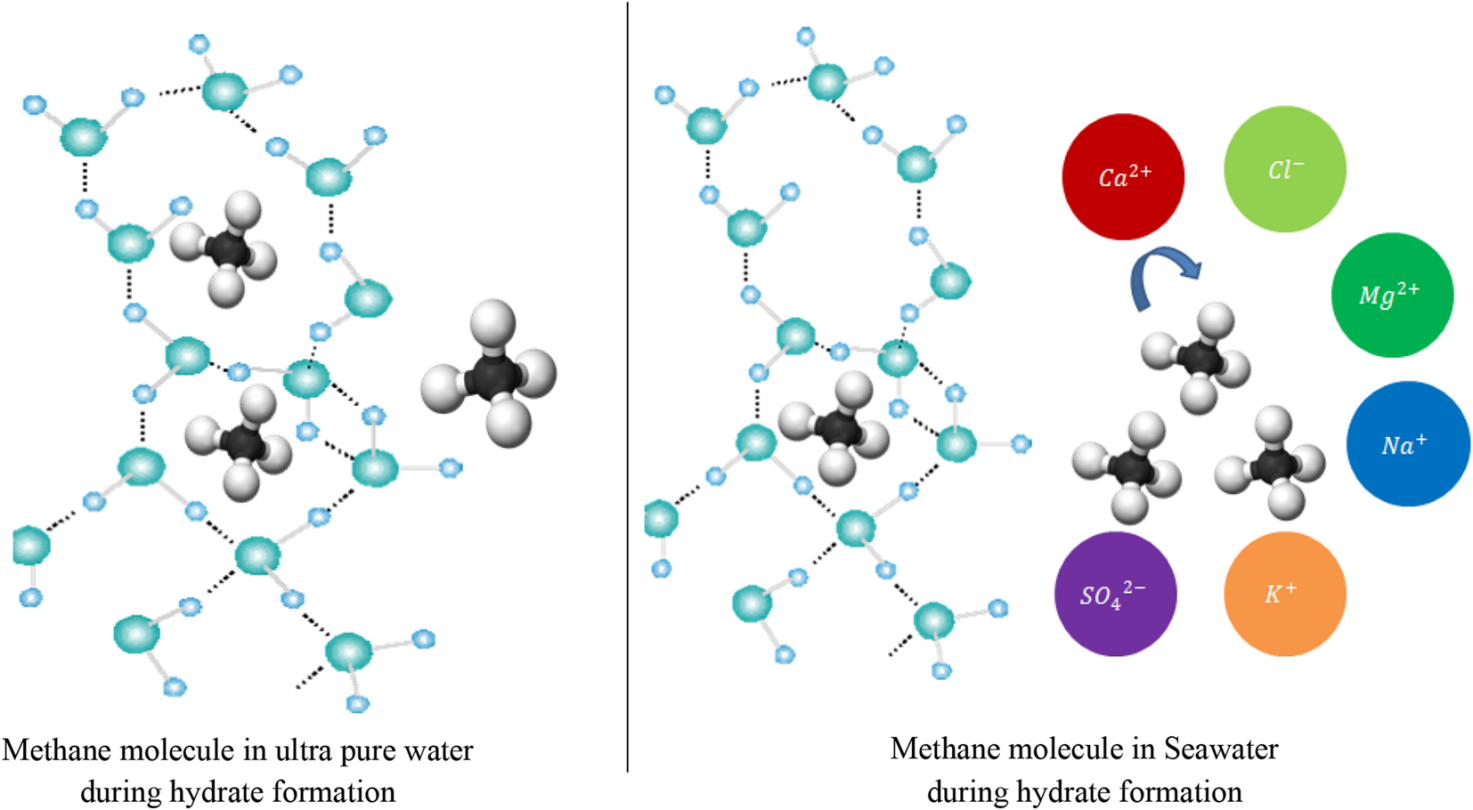
The hypothetical illustration of the hydrate formation in the ultra pure water system and the saline water system in the absence of inhibitor.

In this work, the CH_4_ hydrate formation kinetics were observed in both ultra pure water and Qatar seawater systems. The hypothetical illustration of CH_4_ hydrate formation in the ultra pure water and saline water system is shown in Fig. 8. The ions present in the seawater system may affect the activity of water and gas molecules in aqueous solution and are likely to tend to interact with the gas molecule^68^. This likely makes it difficult for the water molecules to encapsulate gas molecules and form a stable hydrate crystal lattice. The dissolved ions in seawater may interact with the water molecular structure via Columbic forces, which may hinder the formation of the gas hydrates. The presence ions may also reduce the availability of free water molecules in the system and may cause electrostatic interactions with the molecules, impeding the formation of gas hydrates^70,71^.

**Figure 9:**
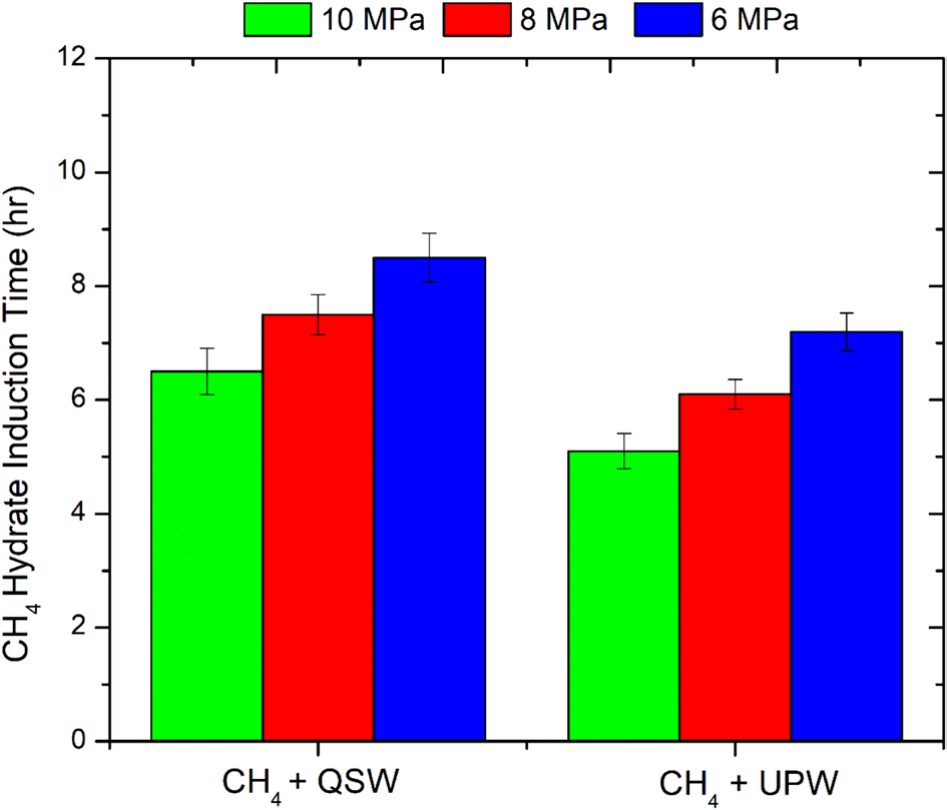
CH_4_ hydrate induction time in the Qatar seawater (QSW) and ultra pure water (PW) system at different pressure conditions. The above data has an uncertainity of about ± 0.33 h.

As shown in Fig. 9, the hydrate induction occurs faster in the ultra pure water system compared to the seawater system. Hydrate induction occurs faster at higher pressures conditions compared to lower pressure conditions. At 6 MPa, the hydrate induction time in ultra pure water system was about 7.2 ± 0.3 h and in Qatar seawater system it was about 8.5 ± 0.3 h. Similarly, at 10 MPa, the hydrate induction time in ultra pure water system was about 5.1 ± 0.3 h and in Qatar seawater system it was about 6.5 ± 0.3 h. As mentioned above, the delay in hydrate induction time in Qatar seawater system may be due to the presence of the ions in the seawater system that may be interacting with the CH_4_ molecule and causing re-orientation of the molecule. As a result, the hydrate formation occurs slower in the seawater system compared to the ultra pure water system. Recently, Thakre et al.^72^ examined the methane hydrate formation dynamics in saline water (1:6). They observed that the presence of salt ions results in vapor–liquid-phase separation. Moreover, due to bubble formation, the two-phase gas–liquid system experiences a low density of methane, which retards the hydrate growth rate. They also stated that for low salt dosage (0.8 wt %) only slight inhibition effect occurs. However, the inhibition effect gets more significant at higher dosages (1.5–5.0 wt %) of salt in water. Idress et al.^73^, also stated that the level of salinity significantly impacts gas hydrate induction time and dissociation due to its ability to act as an inhibitor by delaying the nucleation of methane hydrate. Yang and Xu^43^, highlighted that the thickness of CH_4_ hydrate becomes thinner in saline/ seawater compared to that of the ultra pure water system. Saw et al.^68^, reported that the hydrate dissociation enthalpy decreases with the increase in the temperature and salinity of the synthetic seawater, and the hydrate induction or formation times depends on the sub-cooling temperature and the salt concentration present in the selected c seawater sample.

### KHI effect of Ionic liquids in Pure water and Qatar seawater system

**Figure 10:**
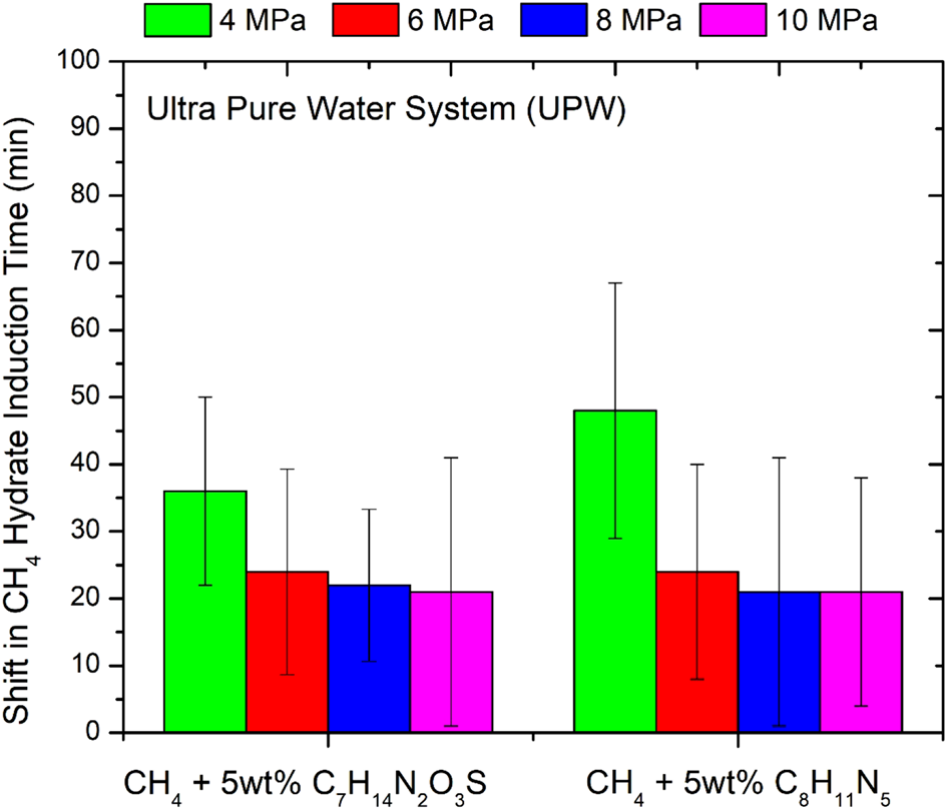
Shift CH_4_ hydrate induction time (ultra pure water system) in the presence of 5wt% IL salts, 3-Ethyl-1-methyl-1H-imidazol-3-ium-methane sulfonate [C_7_H_14_N_2_O_3_S] and 3-Ethyl-1-methyl-1H-imidazol-3-ium-dicyanoazamide [C_8_H_11_N_5_]. The above data has an uncertainity of about ± 0.33 h.

Initially, the kinetic inhibition effect of selected ILs on methane hydrate formation in a ultra pure water system was tested at different pressure conditions (4–12 MPa). As shown in Fig. 10, both ILs showed a slight KHI effect and the methane hydrate induction time was delayed by about 36–48 min (± 20 min) at low pressures (~ 4 MPa) and by 21 min (± 20 min) at high pressures (~ 10 MPa). The maximum time delay of about 48 min (± 20 min) was provided by the IL C_8_H_11_N_5_ at about 4 MPa. In terms of effectiveness as kinetic hydrate inhibitors, the IL C_8_H_11_N_5_ was found to be slightly more effective than IL C_7_H_14_N_2_O_3_S.

**Figure 11:**
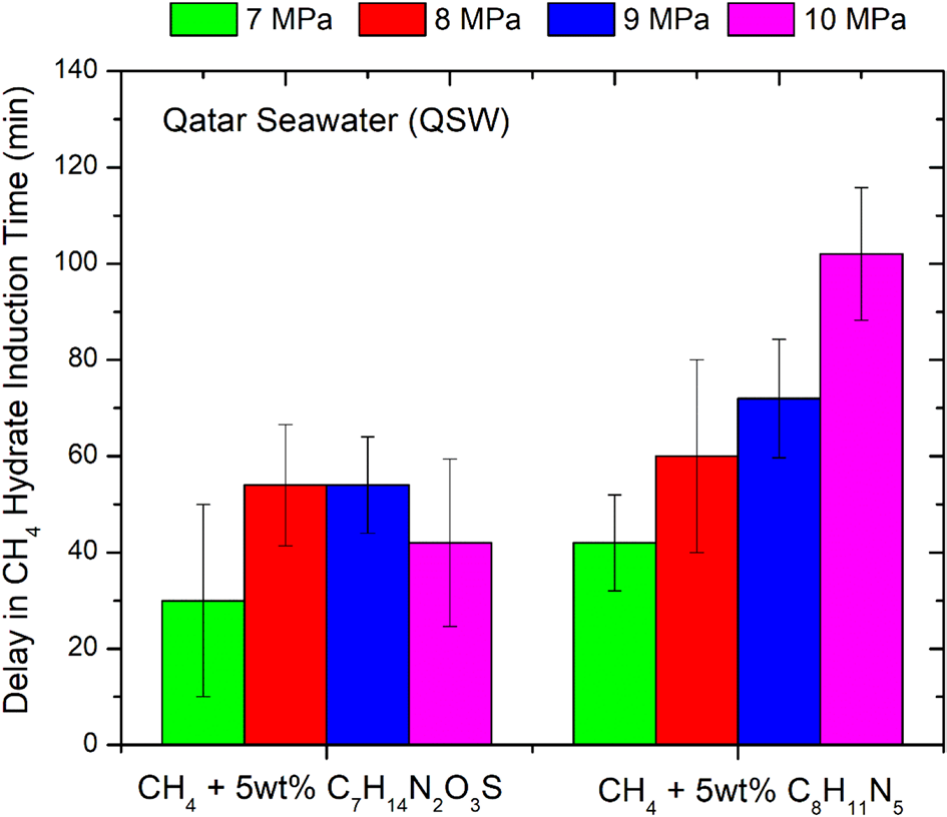
Shift CH_4_ hydrate induction time (Qatar seawater system) in the presence of 5wt% IL salts: 3-Ethyl-1-methyl-1H-imidazol-3-ium-methane sulfonate [C_7_H_14_N_2_O_3_S] and 3-Ethyl-1-methyl-1H-imidazol-3-ium-dicyanoazamide [C_8_H_11_N_5_]. The above data has an uncertainity of about ± 0.33 h.

The kinetic inhibition strength of ILs C_8_H_11_N_5_ and C_7_H_14_N_2_O_3_S in mitigating methane hydrate formation was then tested in the Qatar seawater system (Fig. 11). In the seawater system, both inhibitors performed slightly better than the ultra pure water system. This improvement in the inhibition effect may be attributed to the presence SO_4_^2−^, Cl^−^, Na^+^ and Mg^2+^ ions in the system that may be leading to a synergistic effect. As shown in Fig. 11, the IL C_8_H_11_N_5_ provides better kinetic inhibition effect compared to IL C_7_H_14_N_2_O_3_S again. The maximum time delay of about 102 min (± 20 min) is provided by the IL C_8_H_11_N_5_ at a pressure around 10 MPa. At 7 MPa, both ILs provided a similar time delay of about 30–42 min (± 20 min).

In the above context, the both ILs tend to act as the kinetic hydrate inhibitor and can delay the methane hydrate formation by 21–48 min (± 20 min) in the ultra pure water system and by 30–102 min (± 20 min) in the Qatar seawater system. The presence of the ions SO_4_^2-^, Cl^-^ , Na^+^ and Mg^2+^ ions in the seawater system may be causing a synergistic effect which improves the kinetic inhibition performance of these ILs in the Qatar seawater system.

### Probing hydrate inhibition mechanism in the presence of ILs and methanol

**Figure 12:**
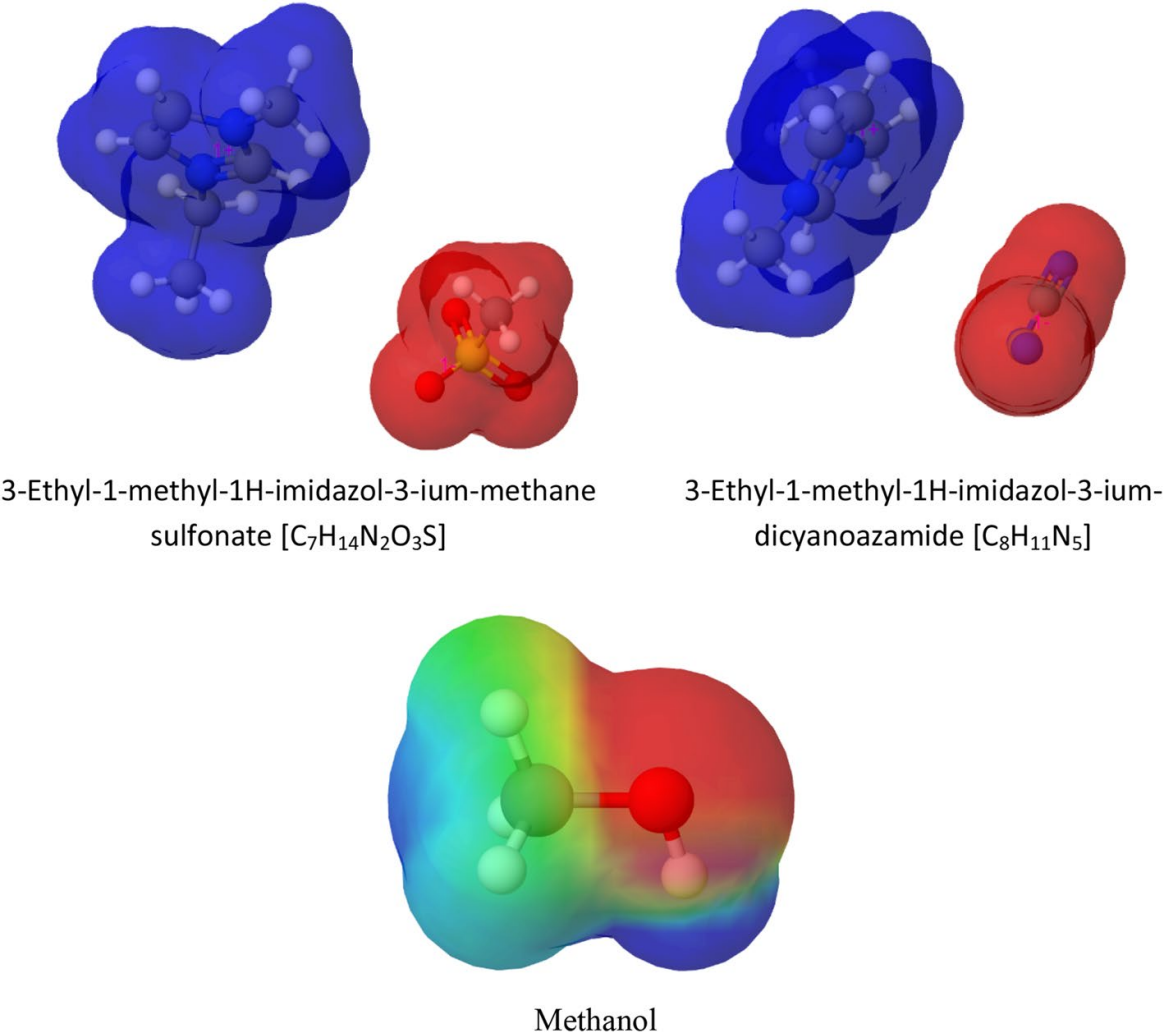
3D computational models of IL salts: 3-Ethyl-1-methyl-1H-imidazol-3-ium-methane sulfonate [C_7_H_14_N_2_O_3_S], 3-Ethyl-1-methyl-1H-imidazol-3-ium-dicyanoazamide [C_8_H_11_N_5_] and Methanol [MeOH].

The 3D molecular models for the Ionic liquids (ILs) salts 3-Ethyl-1-methyl-1H-imidazol-3-ium methane-sulfonate [C_7_H_14_N_2_O_3_S], 3-Ethyl-1-methyl-1H-imidazol-3-ium dicyanoazanide [C_8_H_11_N_5_] and methanol [CH_3_OH] were generated using the Molview^74^. As depicted in Fig. 12, the regions colored in red are the areas of high electron density (-) and the regions colored in blue are the areas of low electron density ( +). The disruption of the hydrogen-bonding network within the hydrate clusters occurs due to the strong electrostatic force of interaction between the ILs and the water molecules. Xiao et al.^75^, also stated that the ILs salts have strong electrostatic charges, and simultaneously their anions/cations may form hydrogen bonding with water disrupting the formation of gas hydrates. They also stated that for ILs with the same anion, the THI effect of ILs with shorter alkyl chain substituent is superior to ILs with longer alkyl chain substituent. The THI effectiveness of ILs may also rely on electrical conductivity (EC) of ILs in sample solutions. ILs with higher EC in sample solutions may exhibit higher THI effects. However, it’s hydrogen bonding strength of ILs with water that is likely to play a significant role in THI effectiveness of selected ILs.

The molecular electrostatic maps in Fig. 12, facilitates the conception of electron density within the ILs and methanol to spot the regions of high and low electron density. An electrostatic potential map in Fig. 12 depicts that the high electron regions (red color) within the IL are the methane-sulfonate and dicyanoazanide ions. As mentioned above, these ions are likely to disturb the hydrogen bonding within water molecules, and cause a strong electrostatic force of interaction with water molecules, interrupting the hydrate formation process. The methanol has -OH groups, as shown in Fig. 12, which may help it to provide a better THI effect compared to the ILs. The strong interfacial-interactions of the –OH group with the hydrogen bonds in the hydrate structure may cause strong disruption or disorientation within the hydrate crystal structure. Vojta and Vazdar^76^, also stated that the crucial element that aids methanol in exhibiting better THI effect is the existence of the –OH group. The strong interface of –OH group with the hydrogen bonds in hydrates and further interface of –CH_3_ group with the C–C or hydrogen bonds within hydrates tend to cause disruption of hydrate crystals^76^. The presence of –OH group is may also play a key role in making hydrogen bonds with water molecules and shifting the thermodynamic equilibrium through hydrogen bonding.

## Conclusions

In this work, the CH_4_ HLVE data in ultra pure water system was compared with CH4 HLVE data in artificial seawater (ASW), Qatar seawater (QSW), and other saline water systems reported in the literature. The CH_4_ pure water HLVE curve was suppressed by about 3 K in Qatar seawater and 2 K in artificial seawater. The hydrate inhibition performance of the Ionic liquids (ILs) salts C_7_H_14_N_2_O_3_S and C_8_H_11_N_5_ was evaluated in the ultra pure water system and Qatar seawater systems at different pressure conditions (4–12 MPa) and their performance was compared with industrial thermodynamic inhibitor methanol and other ILs salts reported in the literature.

The selected ILs exhibited poor hydrate inhibition effect in the ultra pure water systems, but they show a noticeable thermodynamic and kinetic hydrate inhibition effect in the Qatar seawater system. The IL salt C_8_H_11_N_5_ exhibited slightly better hydrate inhibition performance than the IL C_7_H_14_N_2_O_3_S. However, in comparison to methanol, the hydrate inhibition provided by IL salts was not significant in the ultra pure water system and Qatar seawater system both. The better performance of IL salts in Qatar seawater indicates that the presence of dissolved salt ions in the seawater like SO_4_^2−^, Cl^−^, Na^+^ and Mg^2+^ ions helps to improve the thermodynamic inhibition effect of ILs and may lead to a synergistic effect. This work indicates that developing a hydrate mitigation strategy merely on the HLVE data set obtained using a pure water sample is not adequate and actual offshore seawater samples need to be considered. The selected ILs in this work do not exhibit a significant THI effect in comparison to methanol. However, they can delay the hydrate induction time, a benefit that the conventional THI does not offer. This can be considered a step forward in the search of better THI that can be tailored according to the process required and at the same time are environmentally benign. In the future, similar work needs to be conducted using the actual Qatar natural gas mixture and actual offshore Qatar seawater sample. This will help to get a more reliable set of data for the development of a hydrate mitigation strategy for offshore flow assurance.
